# Re-evaluating the risk factors for radiation pneumonitis in the era of immunotherapy

**DOI:** 10.1186/s12967-023-04212-5

**Published:** 2023-06-07

**Authors:** Feihu Chen, Jiling Niu, Min Wang, Hui Zhu, Zhijun Guo

**Affiliations:** 1 Department of Radiation Oncology, Shandong Cancer Hospital and Institute, Shandong First Medical University and Shandong Academy of Medical Sciences, 440 Jiyan Road, Jinan, 250117 Shandong China; 2 Department of Intensive Care Unit, Shandong Cancer Hospital and Institute, Shandong First Medical University and Shandong Academy of Medical Sciences, 440 Jiyan Road, Jinan, 250117 Shandong China

**Keywords:** Radiation pneumonitis, Radiotherapy, Risk factors, Chemotherapy, EGFR-TKI, Immunotherapy, Anti-angiogenesis

## Abstract

As one of the common complications of radiotherapy, radiation pneumonia (RP) limits the prognosis of patients. Therefore, better identifying the high-risk factors that lead to RP is essential to effectively prevent its occurrence. However, as lung cancer treatment modalities are being replaced and the era of immunotherapy has arrived, literature that reviews the parameters and mode of radiotherapy, chemotherapy drugs, targeted drugs and current hot immune checkpoint inhibitors related to RP is lacking. This paper summarizes the risk factors for radiation pneumonia by retrieving and analysing previously published literature and the results of large clinical trials. The literature primarily included retrospective analyses, including clinical trials in different periods and a part of the literature review. A systematic literature search of Embase, PubMed, Web of Science, and Clinicaltrials.gov was performed for relevant publications up to 6 Dec. 2022. Search keywords include, but are not limited to, “radiation pneumonia”, “pneumonia”, “risk factors”, “immunotherapy”, etc. The factors related to RP in this paper include physical parameters of radiotherapy, including V_5_, V_20_, and MLD; chemoradiotherapy mode and chemotherapy drugs, including paclitaxel and gemcitabine; EGFR-TKI; ALK inhibitors; antiangiogenic drugs; immune drugs and the underlying disease of the patient. We also introduce the possible mechanism of RP. In the future, we hope that this article not only sounds the alarm for clinicians but also helps to identify a method that can effectively intervene and reduce the occurrence of RP, significantly improve the quality of life and prognosis of patients, and more effectively improve the therapeutic effect of radiation therapy.

## Background

**Fig.1:**
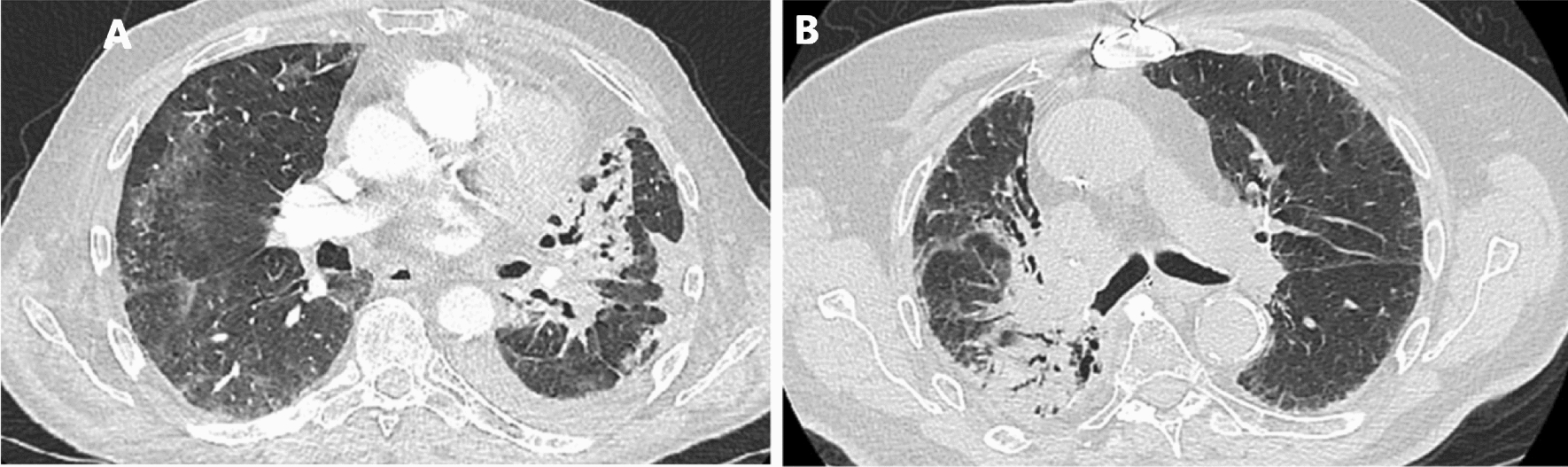
CT image of RP **A**: Axial image from a CT angiogram performed 5 months after radiotherapy demonstrating consolidation and atelectasis consistent with radiation pneumonitis and fibrosis. **B**: The image demonstrates airspace consolidation in the medial right middle lobe and superior right lower lobe with traction bronchiectasis and reticulation within the radiation field, consistent with evolution of radiation fibrosis

Radiotherapy is used consistently throughout lung cancer treatment. Radiation pneumonitis (RP) is a common complication of thoracic radiotherapy. According to the relevant literature, the incidence rate of RP is approximately 30% [1]. RP occurs when normal lung tissue in the radiation field is affected by passing radiation, which causes alveolar exudation inflammation. It is typically marked by dyspnoea, dry cough, hypoxemia, and low fever. Pathological anatomy shows alveolar septal oedema, endothelial cell swelling, vascular wall thickening, and other changes [2, 3]. The main manifestations of CT are patchy and uniform flocculent blurred shadows in the radiological field, accompanied by thickened blood vessels and bronchial shadows and indistinct boundaries with surrounding normal lung tissue. The spots in the radiological area are solid, with a higher density than ground glass opacities, clear edges, and clear boundaries with normal tissue [4]. We attach CT images to demonstrate the above features (Fig. 1).

The interval between the onset of RP and radiotherapy varies in length. Acute RP usually occurs within a few days after irradiation, while most chronic RP occurs within the first 12 weeks to 6 months after radiotherapy [5]. Once RP occurs, it causes irreversible fibrous lesions and even fibrosis of the lung tissue, damages respiratory function, and seriously influences the quality of life and survival of patients.

The risk factors were evaluated in the era of chemotherapy. Regimens such as gemcitabine, patients with diabetes, and the V_20_ of lung dose ≥ 30% have definite relationships with the occurrence of RP. To date, with the advent of immunotherapy, the risk factors for RP urgently need to be re-evaluated to avoid serious events and improve overall efficacy.

## Physical parameters of radiation therapy

### The doses of the opposite mediastinum

A previous retrospective analysis by Takayuki Mikimoto et al. showed that a radiation dose to the opposite mediastinum greater than 40 Gy could be a risk factor for severe RP [6]. The possible reason for this relationship is that an irradiation dose greater than 40 Gy may cause a larger radiation field that includes the contralateral mediastinum. Second, contralateral lymphatic outflow, which is more vulnerable to damage than normal lung tissue and more prone to inflammatory cell infiltration and tissue damage, may be damaged [6].

### DVH

The dose-volume histogram (DVH) parameters of radiotherapy mainly include the mean lung dose (MLD) and V_5_(Percentage of lung volume exposed to radiation greater than 5 Gy to total lung volume), V_10_(Percentage of lung volume exposed to radiation greater than 10 Gy to total lung volume), V_20_(Percentage of lung volume exposed to radiation greater than 20 Gy to total lung volume), V_30_(Percentage of lung volume exposed to radiation greater than 30 Gy to total lung volume) [7]. The occurrence and severity of RP are often closely related to the lung volume that exceeds the lung radiation tolerance threshold. When the entire lung is irradiated, the threshold of RP can be as low as 7 Gy, but when part of the lung is irradiated, the threshold of RP can be as high as 20–30 Gy [8]. In a univariate statistical analysis of RP during concurrent chemoradiotherapy conducted by Tsujino et al., all DVH parameters were related to the occurrence of ≥ grade 3 RP [7].

#### V_20_

In clinical radiotherapy for lung cancer, V_20_ is typically used as a parameter to evaluate the treatment plan. Many studies have reported that V_20_ can be invoked as an independent risk factor for RP. A retrospective analysis published by Graham MV et al. [9] confirmed V_20_ as a risk factor for RP. The results showed that the incidence of RP (above grade 2) was 0.7% and 36% two years later when V_20_ was less than 22% and more than 40%, respectively. At the same time, Tsujin [10], Leprieur [11], Pinnix [12], Farr [13], and other studies confirmed that the incidence of RP increased with increasing V_20_. Therefore, V_20_ is strictly limited in the synchronous chemoradiotherapy mode. A multivariate statistical analysis of RP during concurrent chemoradiotherapy conducted by Tsujino [7] showed that V_20_ ≥ 26% was a risk factor for RP of grade 3 or above. Finally, a meta-analysis by Zhang [14] et al. showed that RP often occurred in patients receiving radiotherapy with V_20_ > 25%.

#### V_5_, V_10_, V_30_

With the development of radiotherapy technology, intensity-modulated radiation therapy, volume arc intensity-modulated radiotherapy technology, and other technologies have emerged, and the lung volume of patients receiving low-dose radiation has increased accordingly [12]. Studies have confirmed that the volume of lungs exposed to low-dose radiation is also associated with RP. Pinnix et al. [12] showed that when MLD ≥ 13.5 Gy, V_5_ ≥ 55%, V_10_ ≥ 40%, V15 ≥ 35%, V_20_ ≥ 30%, and V_25_ ≥ 23%, the occurrence of RP significantly correlated with DVH parameters.

##### V_5_

V_5_ > 55% is an important predictor of RP after detecting the discrete multivariable model. According to the relevant literature, when V_5_ ≤ 42%, the risk of 3 or more radioactivity is 3%, while when V_5_ > 42%, the risk of RP can be as high as 38% [15].

##### V_10_

In a retrospective analysis by Yorke et al. [16], lung V_10_ was proposed to have more advantages than V_20_ in predicting acute RP of grade 3 or above. Shi et al. [17] confirmed that V_10_ (P = 0.015) is the most significant risk factor for severe acute RP.

##### V_30_

Marks B of Duke University [18] proposed in a prospective analysis that the size of V_30_ was significantly positively correlated with the incidence of RP. When V_30_ < 18%, the risk of RP was shown to be extremely low, while when V_30_ ≥ 18%, the risk of RP can reach 24% [19].

#### MLD

Compared with other DVH parameters, MLD has more correlations in predicting RP. MLD refers to the average dose of exposure to the whole lung. MLD and V_20_ have been widely accepted as significant risk factors for the occurrence of RP [20, 21]. Many articles have reported that an increase in MLD also increases the incidence of RP. These two indicators are also used as predictors of RP. According to the American Cooperative Organization for Cancer Radiotherapy's research report, MLD must be limited to ≤ 20 Gy to control the incidence of RP below 20% [19]. A retrospective analysis published by KwaSIS et al. [22] in 2008 that included 540 patients with chest tumours confirmed that MLD was an essential indicator for forecasting RP. Specifically, the value of MLD directly correlated with the incidence of RP in research published by Hernando et al. [19]. In earlier years, the incidence of RP was only 10.0% when the MLD was lower than 10 Gy, while it increased to 44.5% when the MLD was greater than 30 Gy, and this difference was significant. Additionally, Leprieur [11], Zhang [14], Pinnix [12], and others have published many articles confirming that MLD has a high positive correlation with RP.

#### NTCP

The normal tissue complication probability (NTCP) refers to the likelihood of irreversible damage to normal tissue under a precise radiation dose. It mainly depends on the total amount of exposure, the split dose, and the volume of exposure of normal tissues. In a prospective study by Marks LB et al. [23], univariate analysis showed a significant correlation between NTCP and the occurrence of RP.

Martel PC et al. [24] examined 42 lung cancer patients who received 3D-CRT (3-dimensional conformal radiation therapy) radiotherapy without surgery. All patients received radiotherapy doses higher than 67 Gy. The results showed that the average NTCP value in the group with RP was 73% and that in the group without RP was 25%. In addition, Oetzel PC et al. [25] published a paper that included 46 NSCLC (non-small cell lung cancer) patients receiving 3D-CRT. The results were similar to the above: the mean NTCP of the affected lung in the RP group was significantly higher than that in the non-RP group.

#### Stage summary

In summary, DVH factors have been confirmed to be closely related to the occurrence of RP, and with the increase in MLD, and other parameters, the incidence of RP also increases.

Notably, with the widespread inclusion of immunotherapy, the current consensus on the physical dose standard of radiotherapy was established in the era of chemotherapy, but the toxicity of immune drugs and the damage caused by radiotherapy will inevitably be superimposed. At the 2021 ASCO meeting, an abstract proposed that V_20_ should be limited to less than 25% in combination with immunotherapy to control the incidence of RP and other radiation injuries.

At the same time, a few of the other studies on immunotherapy combined with chemoradiotherapy proposed that the restriction of DVH parameters should be stricter, but related evidence or independent clinical studies are currently lacking. In a retrospective analysis by Xiao Ming, the incidence of RP was increased in patients with a larger planning target volume (PTV) or stereotactic body radiotherapy (SBRT) in both lung lobes using immunotherapy [26].

Therefore, future efforts should explore a definite safe dose segmentation model and evaluate DVH parameters that can benefit patients the most.

## Factors of drugs

### Chemotherapy

#### The difference between different chemotherapy agents

Currently, the commonly used chemotherapy drugs for lung cancer include docetaxel, gemcitabine, pemetrexed, etoposide, paclitaxel, and platinum. The incidence of RP varies among different combinations of chemotherapy drugs. In a retrospective study published by Giroux et al. [27], participating in the neoadjuvant chemotherapy program with gemcitabine significantly increased the risk of acute RP. This point of view has been corroborated by a Phase I clinical trial [28]. The dosage of the drug is strongly related to the incidence of RP. The consequences of single-factor analysis in an article published by Cui Xiaoying et al. [29] showed that the cumulative dose of gemcitabine (> 9.0 g) was 3.45 times higher than that of gemcitabine (< 9.0 g). Chemotherapy regimens containing paclitaxel appear to be more likely to cause RP. In a clinical trial conducted by Liang et al. [30], regimens consisting of simultaneous radiotherapy with etoposide and platinum or paclitaxel and platinum were compared. The results showed that the incidence of RP in the paclitaxel and platinum groups was significantly higher than that in the etoposide and platinum groups. Clinicians should be aware of this finding.

#### The modality of chemotherapy combined with radiotherapy

Chemoradiotherapy is one of the primary treatment modes for lung cancer at present. The administration of chemotherapy drugs includes concurrent chemoradiotherapy and sequential chemoradiotherapy. The incidence of RP varies with different dosing modes. According to Bledsoe et al. [4], Zhang et al. [14] and a large number of other studies, systemic comprehensive treatment with radiotherapy and chemotherapy will increase the sensitivity of lung tissue to radiation compared with radiotherapy alone, thus increasing the incidence of RP [31]. Parashar et al. [32] also indicated in their research that the incidence of RP after concurrent chemoradiotherapy can be increased five-fold compared with radiotherapy alone. The results of a Phase I clinical trial [28] showed that patients are 30% more likely to develop RP when radiotherapy of locally advanced NSCLC is synchronized with gemcitabine.

Sequential chemoradiotherapy appears to be more toxic. A meta-analysis based on previous literature suggests that [33] the risk of RP after sequential radiotherapy is higher than that of concurrent chemoradiotherapy. In addition, a study by Liu Ruifeng and others also confirmed that the incidence of grade 4 and above RP was less in the concurrent chemoradiotherapy group than in the sequential radiotherapy and chemotherapy group [34]. In further studies, the location of pneumonia in sequential chemotherapy and radiotherapy coincided with the radiation field, which may be caused by a radiotherapy recall reaction [35]. Finally, Seppenwolde et al. concluded from their comprehensive studies that although the incidence of RP was increased by concurrent chemoradiotherapy compared with those receiving radiotherapy alone, the risk of severe RP was acceptable [10, 36]. At the same time, the incidence of severe RP was lower with concurrent chemoradiotherapy than with sequential radiotherapy and chemotherapy. Concurrent chemoradiotherapy was preferred based on the tumour control rate, remission rate, and long-term survival rate.

### EGFR-TKI

Epidermal growth factor receptor-tyrosine kinase inhibitors (EGFR-TKIs) have become the first-line treatment for patients with advanced lung adenocarcinoma with EGFR-sensitive mutations [37]. Combined with local complementary radiotherapy, these drugs can significantly improve the survival of patients. At present, many studies confirmed that EGFR-TKIs can not only increase radiosensitivity but also that radiotherapy can reduce EGFR-TKI resistance [38–40]. The incidence of RP also increases.

A retrospective study published last year showed that the combination of EGFR-TKI and radiotherapy significantly increased the incidence rate of mild to moderate RP. Fortunately, the incidence of grade 4–5 adverse events did not significantly change [41]. A retrospective analysis of EGFR-TKIs and radiotherapy for advanced non-small cell lung cancer patients with EGFR mutations showed that the incidence of RP above grade 3 or even fatal RP was as high as 10% [42]. In 2018, Professor Zhou Caicun reported that 7.7% of patients suffered from RP of grade 3 or above when EGFR-TKI maintenance treatment was added to radiotherapy-based local ablation treatment [43]. Professor Sun Jianguo [44] published a clinical study on simultaneous EGFR-TKI and chest radiotherapy for Stage IV NSCLC patients, confirming the above view. Currently, commonly used EGFR-targeted drugs are divided into first-generation drugs represented by gefitinib, icotinib, and erlotinib; second-generation drugs represented by afatinib; and third-generation drugs represented by osimertinib. This section focuses on describing and comparing the occurrence of RP between first-generation and third-generation drugs (Table 1).

**Table 1:** RP of EGFR-TKIs related studies

Drugs	Study	Published year	study type	No. of cases	Radiation dose(Gy)	RP rate(%)
First-generation EGFR-TKIs	Xu K [41]	2021	Retrospective study	45	50–66	37.7
	Chih-Chia Chang [42]	2011	Clinical Trial	25	40–50	84.0 > 3Grade: 12.0
	Caicun Zhou [43]	2018	Retrospective study	145	33–60	> 3Grade: 7.7
	Jianguo Sun [44]	2019	Clinical Trial	10	54–60	40.0 > 3Grade: 20.0
	Wenxiao Jia [49]	2021	Retrospective study	67	50–60	44.78 > 3Grade: 8.9
	C Yong [45]	2016	Clinical Trial	316	40–65	15.1
Erlotinib	Zhuang H [46]	2014	Retrospective study	24	44–46	36.0 > 3Grade: 21.0
Gefitinib	Rothschild S [47]	2011	Clinical Trial	14	63	14.0
	Okamoto I [48]	2011	Clinical Trial	9	60	28.0
Oxitinib	Wenxiao Jia [57]	2021	Retrospective study	11	60	63.6
Crizotinib	Hou H [61]	2019	Meta-analysis	/	/	4.2

*RP* Radiation pneumonitis, *EGFR-TKI* Epidermal Growth Factor Receptor-Tyrosine Kinase inhibitor

#### First-generation drugs

A multicentre collaborative clinical study of a second-line gefitinib/erlotinib combined radiotherapy regimen included 316 NSCLC patients, 106 of whom received gefitinib/erlotinib combined radiotherapy and 210 of whom received monotherapy. The results showed that pulmonary inflammatory changes occurred in 16 patients (15.1%) in the combination group and 21 patients (10%) in the monotherapy group, but the difference between the two groups was not statistically significant, and the combination therapy did not increase the pulmonary toxicity of TKIs alone [45].

##### Erlotinib

First, a prospective study analysed the prevalence of RP in patients with non-small cell lung cancer receiving erlotinib synchronous radiotherapy. The results showed that 37.5% of the patients had grade 2 or above RP, and 8.7% had grade 3 RP. Therefore, the potential development of RP in patients receiving chest radiotherapy and erlotinib treatment simultaneously should be closely monitored [46].

##### Gefitinib

Many small-sample experimental results have shown that the incidence of RP after combined radiotherapy is relatively high for gefitinib. Rothschild et al. reported the results of a multicentre Phase I study of gefitinib combined with chest radiotherapy. Specifically, 2 of the 14 patients (14%) developed grade 2 pneumonia, but no deaths occurred [47]. Okamoto et al. included 9 patients with Stage III unresectable NSCLC. In this study, 7 patients received gefitinib combined with chest radiotherapy, and 2 patients (28%) developed grade below grade 3 pneumonia [48].

In addition, overlap time is an independent risk factor for RP in patients treated with simultaneous EGFR-TKI and thoracic radiotherapy. Jia Wenxiao's retrospective analysis showed that the overlap time of first-generation EGFR-TKIs and chest radiotherapy exceeded 20 days [49]. The chest radiotherapy duration exceeded 32 days and was significantly related to grade 2 and above RP. At the same time, this analysis also showed that the incidence of grade 2 and above RP in NSCLC patients who used first-generation EGFR-TKIs and chest radiotherapy was as high as 44.78%, and 8.96% of these patients had grade 3 RP.

#### Third-generation drugs

Oxitinib represents the third generation of drugs. The mechanism of action of oxitinib is somewhat different from that of previous-generation EGFR drugs. It is an irreversible EGFR inhibitor that can be transcribed in alveolar epithelial cells [50]. While inhibiting EGFR, it also inhibits the proliferation of alveolar epithelial cells and prevents their self-repair in response to radiation damage. Second, oxitinib also reduced G2/M phase arrest in irradiated cells and delayed DNA damage repair, and it could play a role in radiation sensitization. However, it also enhanced radiation damage to normal lung tissue [50, 51]. In addition, earlier AURA series studies and FLUARA studies showed that the incidence of interstitial lung disease with oxitinib was 2%-4%, which was slightly higher than that with first- and second-generation TKIs [52–56]. Jia Wenxiao also showed in a retrospective study that the incidence of RP of grade 2 and above was 63.6% when oxitinib was used at the same time as chest radiotherapy [57].

Given the above findings, patients who utilize EGFR drugs and chest radiotherapy simultaneously should be alert to the occurrence of RP, especially those who use third-generation drugs combined with chest radiotherapy.

Whether EGFR-TKI combined radiotherapy will increase pulmonary toxicity in patients compared with TKI alone warrants examination. Compared with the clinical statistical data of pulmonary toxicity of a single-drug EGFR-TKI that included thousands of cases, most of the combined treatment regiments are small sample studies, and statistical results of large samples for pulmonary toxicity are lacking. Consequently, the incidence of pulmonary toxicity (1–80%) and mortality (approximately 33%) caused by the combined treatment regiments significantly differ.

### ALK inhibitor

Anaplastic lymphoma kinase (ALK) gene mutation, a rare mutation in NSCLC, is addressed by three generations of clinical drugs. The ALK gene mutation is also known as the diamond mutation in NSCLC because of its low mutation rate of only 6–8% [58]. Currently, the main drugs targeting ALK mutations in clinical use include crizotinib in the first generation, ceritinib and alectinib in the second generation, and lorlatinib in the third generation. Both the PROFILE and ALEX studies confirmed that the above-targeted agents could significantly improve the OS and PFS of patients with non-small cell lung cancer with ALK gene mutation compared with standard chemotherapy [58, 59]. The toxic side effects are within the clinically acceptable range. According to the findings above, one of ALK inhibitors' most common adverse effects is interstitial pneumonia, which occurs in approximately 8% of cases. Few studies have been related to RP caused by the combination of chest radiotherapy. However, several case studies still report that ALK inhibitors combined with chest radiotherapy significantly increase the incidence of pneumonia [60, 61]. Therefore, we suggest that although the incidence of ALK inhibition-associated pneumonia is low, clinicians should still be sufficiently vigilant. (Table 1).

### Antiangiogenic drugs

**Fig.2:**
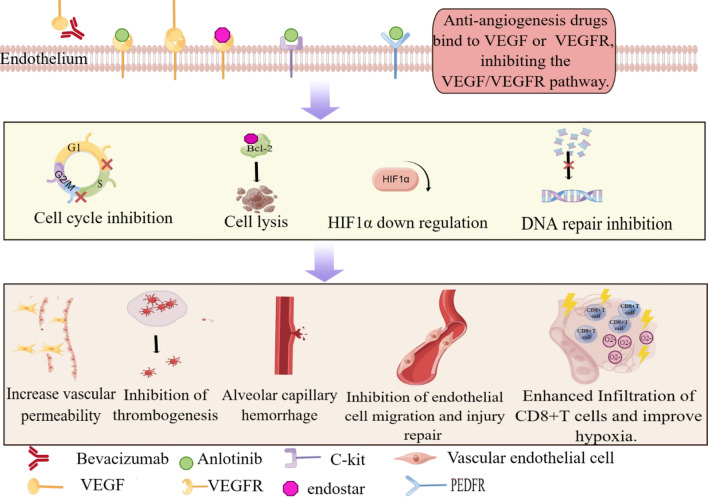
Toxicity overlay of anti-angiogenesis drugs combined radiotherapy. Bevacizumab acts on VEGF, blocking its binding to VEGFR. Anlotinib and endostar competitively bind to VEGFR, similarly inhibiting the VEGF/VEGFR pathway. Anlotinib can also bind to multiple receptors such as PEDFR, and C-kit. Through the above binding process, various cell activities mediated by the corresponding pathway can be inhibited: (1) cell cycle inhibition(main parts); (2)act on intracellular Bcl-2 protein to cause cell lysis; (3) downregulate HIF1α Factor; (4) inhibits DNA repair. Ultimately leading to pathological changes: (1) increased vascular permeability; (2) inhibits platelet production; (3) alveolar capillary hemorrhage (4) inhibits endothelial cell migration; (5) improving the radiosensitivity of tumor cells; (6) recruiting CD8 + T cells to infiltrate the tumor microenvironment to aggravates the inflammatory response. The above series of changes make patients receiving anti-angiogenesis drugs and chest radiation more prone to pulmonary fibrosis and exudation, ultimately leading to an increased risk of radiation pneumonia

One of the critical factors of tumour growth is tumour angiogenesis, and the essential receptor of tumour angiogenesis is the angiogenesis factor vascular endothelial growth factor (VEGF) [62]. Therefore, we can inhibit tumour angiogenesis by inhibiting VEGF, thereby inhibiting tumour growth. In addition, tumour cells cannot effectively supply oxygen and nutrients to tumours due to rapid proliferation and the abnormal structure and function of new blood vessels; therefore, hypoxia is common [63, 64]. However, severely hypoxic cells are not sensitive radiotherapy [65]. Anti-VEGF can improve the hypoxic condition of cells by inhibiting angiogenesis, thereby increasing the sensitivity of tumour cells to radiotherapy. At the same time, due to the inhibition of angiogenesis, the ability of normal lung tissue to address radiation injury and repair will also decline, and the incidence of radiation-induced lung injury will also increase. Extensive literature and case reports indicate that combining antiangiogenic drugs and radiotherapy dramatically increases the incidence of radiation pneumonia. In an analysis of the toxic effects of antiangiogenic drugs combined with SBRT in the treatment of central lung cancer, the observed incidences of grade 3 or higher toxicities (n = 19, 22% of 88 patients) and fatal toxic events (n = 10, 11% of 88 patients) were relatively high [66]. Currently, commonly used antiangiogenic drugs mainly include bevacizumab, endostar, and anlotinib. All three drugs increase the risk of RP by inhibiting the damage repair pathway of endothelial cells and recruiting more inflammatory factors. We have summarized the mechanism in Fig. 2.

#### Bevacizumab

VEGF is the main pathway of vascular endothelial cell proliferation. In tumour tissue, the tumour blood vessels are disordered and distorted due to rapid growth. Consequently, tumour tissue is consistently in an oxygen-poor environment, which causes tumour vascular endothelial cells to secrete more VEGF, creating a vicious cycle. As free radicals, oxygen molecules can stabilize the damage caused by radiotherapy while also affecting the cell cycle arresting cells in the G0 phase.

Therefore, oxygen-poor cells are not sensitive to radiotherapy. Bevacizumab is a monoclonal antibody against VEGF. It can accurately and efficiently bind VEGF, thus inhibiting binding to the VEGF receptor (VEGFR). On the one hand, it can normalize tumour blood vessels, improve the hypoxic environment of tumour tissues and sensitize them to radiation. On the other hand, VEGF antagonism affects VEGF-mediated vascular injury repair, leading to the blocked migration of injured vascular endothelial cells, increased vascular permeability, small artery and capillary bleeding, thus recruiting more inflammatory factors locally and exacerbating the risk of pneumonia [67].

Therefore, the safety of the combination of bevacizumab and chest radiotherapy is unacceptable due to the normal vasotoxicity of the drug itself and the average tissue-killing effect of radiation. Six patients treated with radiotherapy combined with bevacizumab were included in a small sample clinical trial. A total of 5 patients had RP, and two had RP above grade 3 [68]. Previously, many clinical trials and related research analyses showed that the severity of adverse reactions to bevacizumab combined with chest radiotherapy was unacceptable. RP, tracheoesophageal fistula and haemoptysis incidence exceeded the clinically acceptable range. Many clinical trials were interrupted due to the high severity and incidence of adverse reactions; therefore, the two drugs should not be used together [68–71].

#### Endostar

Endostar acts on VEGFR, which blocks the binding of VEGF and endothelial cells, thus causing VEGF to lose its role in mediating neovascularization. Moreover, Endostar can also downregulate the mRNA and protein expression levels of VEGF, thus preventing VEGFR from signal transduction, which inhibits the proliferation and migration of vascular endothelial cells as well as the activation of vascular endothelial cells. Consequently, cells enter a stagnant state, which ultimately inhibits the development and metastasis of tumours. In addition, Endostar can also sensitize cells to radiotherapy by downregulating the level of the hypoxia-inducing factor HIF-1, blocking the cell cycle in G2/M phase, and blocking DNA repair and other mechanisms. Finally, endostar will recruit more CD8 + T cells and macrophages to infiltrate the tumour microenvironment. Therefore, the toxicity of Endostar combined with radiotherapy is due to the blockade of repair and the infiltration of inflammatory factors [72].

The safety of Endostar combined with chest radiotherapy is acceptable. Similar to bevacizumab, Endostar can also affect vascular repair in normal tissue damaged by radiation, but Endostar is a multitarget inhibitor with much fewer toxic side effects than bevacizumab. In a retrospective analysis published by Zhang et al. in 2020, adding Endostar to chest radiotherapy did not significantly increase major adverse events. The incidence of grade 3 or above RP was 10.9% [73]. Many studies confirmed that the incidence of pneumonia and other related adverse events was significantly lower for the combination of Endostar and chest radiotherapy than for the combination of bevacizumab and chest radiotherapy and was within the acceptable range of clinical treatment. The HELPER Study was a prospective clinical study conducted in 2019 on Endostar combined with concurrent chemoradiotherapy to treat Stage III NSCLC patients. The analysis showed that the combination of Endostar and chest radiotherapy resulted in toxic and side effects similar to those in other extensive studies on the incidence of RP, indicating that this treatment was safe and acceptable. The incidence of grade 2 or higher RP was only 11.9%, and grade 5 or higher RP was observed in only 1 of 67 enrolled patients [74].

#### Anlotinib

Anlotinib is a small-molecule multitarget tyrosine kinase inhibitor developed in China. It acts mainly by blocking VEGFR1-3, fibroblast growth factor receptor (FGFR1-4), stem cell factor receptor (c-Kit), and other multitarget pathways, ultimately inhibiting antitumour angiogenesis and tumour proliferation and invasion [75]. In addition, anlotinib can also act on tubulin to inhibit the formation of spindles and arrest cells in the G2/M phase, which not only enhances radiation sensitivity but also affects radiation damage repair and ultimately exacerbates inflammation [76].

The incidence of adverse events of anlotinib was between the two. In the analysis of ALTER0303 and its subgroups, the incidence of adverse reactions related to vascular damage, such as haemoptysis, was low, with only 2% of patients with adenocarcinoma and squamous cell carcinoma occurring in approximately 10% of patients. However, the incidence of adverse vascular reactions, such as haemoptysis, seems to improve significantly after combining treatment with chest radiotherapy [77, 78]. A small-sample study showed that among seven patients who completed both anlotinib and radiotherapy and chemotherapy and then entered the consolidation phase, four patients developed RP, and the incidence of treatment-related fatal adverse events was 28.57%, including fatal pneumonia and haemolysis [79].

In addition, the interval is also an essential factor affecting the incidence of vascular injury-related adverse reactions. In April 2019, Wang et al. found that compared with other patients, the incidence of fatal pulmonary haemorrhage was higher in patients with a history of anti-angiogenesis drug use within 90 days (receiving SBRT treatment) [66].

#### Stage summary

Endostar can be used as an antiangiogenic drug in combination with radiotherapy, and anlotinib can also be used. Nevertheless, patients need to be closely observed and clinicians should be alert to the occurrence of adverse reactions. Because of the high incidence of adverse events, bevacizumab is not recommended for use as an antiangiogenic drug in combination with chest radiotherapy. However, antiangiogenic therapy and local radiotherapy clearly exert a significant clinical benefit to patients. Therefore, we maintain that antiangiogenic therapy should not be abandoned for Stage III NSCLC. Although attempts to combine antiangiogenic therapy with chest radiotherapy have consistently failed, the combined treatment of antiangiogenic drugs and chest radiotherapy should not be ignored entirely. We have not identified the best application mode for this combination.

Notably, the three antiangiogenic drugs combined with radiotherapy are not the same. The incidence of RP was higher for bevacizumab than for anlotinib (66.6% vs. 57%). Nevertheless, data to show the difference are scarce due to the small number of patients enrolled in related clinical trials. The incidence of RP at for Endostar as described above is acceptable (Table 2). We believe the additional safety concerns of the above three drugs is due to several reasons. First, as a monoclonal antibody, bevacizumab directly blocks the VEGF receptor efficiently and accurately, thus playing an antiangiogenic role. Nevertheless, the VFGF receptor in normal tissues will also be inhibited, and repair after radiation damage will be blocked. Anlotinib is a small-molecule and multitarget tyrosine kinase inhibitor with a more robust mechanism of action than a monoclonal antibody. Nevertheless, the safety of combined radiotherapy is relatively poor due to its wide range of targets and poor selectivity, affecting multiple pathways, including VEGF and FGFR (fibroblast growth factor receptors). Finally, Endostar is different from the above drugs in that it is endogenous endostatin, which only acts on the vascular endothelium and achieves antiangiogenic effects by inhibiting the advance of endothelial cells. In addition, its half-life is short, and its action is relatively mild. Currently, detailed studies have not confirmed and compared the incidence of pneumonia of the three drugs combined with radiotherapy and its causes. We hope to explore the different mechanisms of these three types of medications on the generation of radiation pneumonia in subsequent studies.

**Table 2:** AEs of anti-angiogenesis drugs related studies

Drugs	Study	Published year	study type	No. of cases	Radiation dose(Gy)	AEs rate(%)
Bevacizumab	Wang C [66]	2019	Clinical Trial	88	84	> 3Grade: 22 fatal events:11
	Lind.J S [68]	2012	Clinical Trial	6	66	RP:83 > 3Grade: 33
	Swog S0533 [71]	2015	Clinical Trial	29	64	> 3Grade: 9.0 fatal events:9.0
Endostatin	Zhang S L [73]	2020	Meta-analysis	271	60–66	RP: ECRT:10.9;ERT:9.4%RE: ECRT:11.6;ERT:12.2
	Zhai Y (HELP study) [74]	2019	Clinical Trial	73	60–66	RP:11.9 > 3Grade: 58.2 5Grade: 2
Anlotinib	Baohui H (ALTER 0303) [77]	2018	Clinical Trial	296	/	Hemoptysis ACC:1.3;SCC10.6
	Zhu H [79]	2022	Clinical Trial	7	54–66	RP:83 fatal events:28.57

*RP* Radiation pneumonitis

### Immunotherapy

The treatment of lung cancer changes with each passing day. With the arrival of nivolumab and pembrolizumab, lung cancer treatment has gradually entered the era of immunotherapy. At the same time, the data from the IMpower-133, PACIFIC, KEYNOTE-799, and many immunotherapy drug studies have also promoted the use of immunotherapy. Compared with previous radiotherapy and chemotherapy, immunotherapy can significantly improve the OS and PFS of patients. Currently, the maintenance of immune consolidation after radical concurrent radiotherapy and chemotherapy has been written into the 2021 CSCO guidelines [80]. However, while immunotherapy benefits patients, we should also heed the accompanying adverse reactions. In addition to the most common dermatitis and asthenia, immune-associated pneumonia is an adverse reaction that needs careful attention. Combined therapy with radiotherapy will increase the incidence of RP. Multiple clinical studies have shown an overlap in toxic side effects between immunotherapy and RT (radiation therapy) [81]. Many studies have confirmed that immune maintenance therapy after concurrent chemoradiotherapy for locally advanced NSCLC can significantly benefit patients. Nevertheless, the incidence of grade 3–4 RP in the immunotherapy group also increased (4.4% and 3.8%). In addition, patients who received immunotherapy had a significantly higher risk of more severe pneumonia than those who received radiotherapy alone [82, 83].

#### Possible mechanism

On the one hand, RT can cause DNA damage to normal lung tissue, causing alveolar capillary epithelial cells to recruit cytokines that activate immune cells and myofibroblasts, which can induce the activation and recruitment of more T cells and enhance the infiltration of local T lymphocytes. On the other hand, immunotherapy can effectively restore the baseline T-cell immune response against tumours. Activated T cells secrete inflammatory cytokines that recruit more immune cells, which amplifies the inflammatory response in irradiated normal tissue and leads to excess immune cell infiltration and the release of inflammatory cytokines, enhancing pulmonary toxicity [84, 85].

Currently, the immune drugs commonly used in clinics mainly target PD-1 (programmed cell death 1 inhibitor) and PD-L1 (programmed cell death-ligand 1 inhibitor). PD-1 inhibitors mainly include tislelizumab, pembrolizumab, nivolumab, sintilimab, and camrelizumab, while PD-L1 inhibitors mainly include atezolizumab and durvalumab. The different mechanisms and correlations related to the occurrence of RP are described separately below.

#### PD-1 inhibitors

Compared with PD-L1 inhibitors, PD-1 inhibitors were associated with a slightly higher incidence of pneumonia, mainly grade 3–4 pneumonia (1.1% vs. 0.4%) [86]. A meta-analysis showed that the incidence of pneumonia of any grade in NSCLC patients treated with PD-1 inhibitors was 4.1% [87]. One of the possible mechanisms for the higher incidence of pneumonia with PD-1 inhibitors lies in blocking the interaction between PD-L2 (programmed cell death-ligand 2 inhibitors) and PD-1 through PD-1. This blockade facilitates cytokine release and the proliferation of autoreactive T cells. As a result, T cells in lung tissue are strongly cloned and amplified, exacerbating the inflammatory reaction [88].

Botticella et al. evaluated the incidence of pneumonia in 318 patients with non-small cell lung cancer who had received previous chest radiotherapy and second-line immunotherapy in a retrospective analysis. The results showed that PD-1 inhibitors significantly increased the risk of pneumonia in patients [89].

KEYNOTE-001 studied the safety of pembrolizumab, a representative PD-1 inhibitor, in the treatment of non-small cell lung cancer patients [90]. The secondary analysis of the study compared the progression-free survival (PFS), overall survival (OS), and pulmonary toxicity of patients who received chest radiotherapy before immunotherapy with those of patients who did not receive chest radiotherapy. The results showed that compared with patients who did not receive chest radiotherapy, patients who received chest radiotherapy had a higher incidence of pulmonary toxicity of any grade. Significant pulmonary toxicity occurred in 15 (63%) of the 24 patients who had received chest radiation and 29 (40%) of the 73 patients who had not received chest radiation. The incidence of pneumonia was also higher in patients with prior chest radiation therapy than in patients without initial chest radiation therapy (13% vs. 1%), but the incidence of grade 3 or more severe pulmonary toxicity was similar between the two groups. Compared with sequential immunotherapy with radiotherapy, simultaneous treatment was associated with higher-grade unfavourable reactions (41.6% vs. 24.8%) and pneumonia (7.1% vs. 3.9%) [90].

The LUN14-179 study, a multicentre Phase II single-arm study conducted by Merk aimed to repeat the PACIFIC trial and enrolled 92 patients with unresectable Stage III non-small cell lung cancer receiving consolidation pembrolizumab, which was followed by concurrent chemoradiation for patients. Toxicity results showed a 15.1% incidence of grade > 2 RP [91].

KEYNOTE-799 used pembrolizumab to improve immunotherapy and apply it simultaneously with radiotherapy and chemotherapy to evaluate its efficacy and safety in unresectable Stage III NSCLC. In terms of safety, one of the primary endpoints of KEYNOTE-799 was that the incidence of pneumonia above grade 3 was less than or equal to 10%. The results showed that 16 patients (7.5%) had pneumonia above grade 3, but the incidence of pneumonia above grade 3 met expectations and did not exceed the set threshold, and the safety was controllable [92]. In addition, a single Phase I clinical study of pembrolizumab combined with concurrent chemoradiotherapy showed that the incidence of grade 3–5 pneumonia was 2.0% [93]. This study also evaluated the pulmonary toxicity of other immune drugs, including the incidence of grade 3–5 pneumonia when nivolumab was combined with concurrent chemoradiotherapy, which was 11.7% [94]; atezolizumab combined with concurrent chemoradiotherapy had an acceptable rate of grade 3–5 pneumonia (3.3%) [95].

#### PD-L1 inhibitors

The mechanism by which PD-L1 exacerbates RP is described above, and the incidence of pneumonia is lower than that of PD-1 inhibitors. In the PACIFIC study, durvalumab immunotherapy was consolidated after radical concurrent chemoradiotherapy for patients with Stage III nonresectable NSCLC, and durvalumab served as a representative PD-L1 inhibitor. Compared with previous radiotherapy and chemotherapy alone or combined with targeted drugs, this treatment mode can prolong patient survival, but its safety warrants exploration.

The safety and toxicity of immunotherapy within two months after radiotherapy were evaluated in 713 patients in the Phase III PACIFIC trial. The incidence of pneumonia in the immunotherapy group was markedly higher than that in the radiotherapy group alone (33.9% vs. 24.8%) [83, 96]. In a study conducted by Moore et al., thirty-nine patients with Stage III NSCLC who received sequential radiotherapy and durvalumab were analysed [97]. The results showed that the incidence of grade 2 or above pneumonia was 54%. In addition, a retrospective analysis by Inoue et al. [98]. The adverse reactions of 30 patients with locally advanced NSCLC who received radiotherapy combined with durvalumab treatment showed that among 19 patients with grade 2 or higher pneumonia, 52% received durvalumab treatment. In contrast, 20% of them only received concurrent chemoradiotherapy. In a subsequent multivariate analysis, durvalumab was associated with an increased risk of developing radiation RP at any level [98, 99]. In another study, durvalumab was shown to cause a much higher risk of radiation pneumonia than other immune drugs [100]. However, both the KEYNOTE-001 and PACIFIC studies showed that the incidence rate of pneumonia at all levels was higher in patients receiving combination therapy. However, the risk of developing advanced pneumonia did not increase significantly [101].

Finally, a period of radiotherapy followed by immunotherapy can induce radiation-recalled pneumonia, which is triggered by the process of “memory” and “overreaction” of immunomodulatory action, which is also of concern to clinicians [102].

#### Stage summary

The risk of RP caused by combined immunotherapy and radiotherapy is acceptable. Although the incidence of RP is increased compared with that of concurrent chemoradiotherapy, it only increases the incidence of RP below grade 3. It does not increase the incidence of more severe RP. Therefore, radiotherapy and immunotherapy can be combined safely. In addition, a study published in JAMA Oncology in 2022 shows that the time window of the combination of immunotherapy and radiotherapy is the most important factor. Specifically, a longer interval corresponds to safer treatment. If the interval exceeds 90 days, the incidence rate of RP will not be substantially increased. Although the risk of RP is increased if the interval is shorter than 90 days, the increase in the rate of severe RP is only approximately 1–1.5% [103] (Table 3).

**Table 3:** RP of immunotherapy related studies

Drugs	Study	Published year	study type	No. of cases	Radiation dose(Gy)	RP rate(%)
Durvalumab	Pacific study [83]	2017	Clinical Trial	713	54–66	33.9
	Moore [97]	2020	Clinical Trial	39	60	54.0
	Inoue [98]	2020	retrospective study	30	60–64	73.0 > 3Grade: 0
Atezolizumab	Lin S H [95]	2020	Clinical Trial	52	66	P1:10.0;P2:16.0
Nivolumab	Peters S [94]	2021	Clinical Trial	79	66	> 3Grade: 11.7
ICI + RT	Botticella A [89]	2019	retrospective study	318	50–60	16.7
Pembrolizumab	Verma V [127]	2018	retrospective study	60	50–60	> 3Grade: A:25;B:5;C:36
	Keynote-001 [90]	2017	Clinical Trial	550	/	A:40;B:63 > 3Grade: A:12;B:17
	Durm G A [91] (Lun14-179)	2020	Clinical Trial	93	59.4–66.6	> 2Grade: 15.2
	Keynote-799 [92]	2021	Clinical Trial	216	60	A:17.9;B:7.8 > 3Grade: A:1.8;B:1.0;
	Jabbour S K [93]	2020	Clinical Trial	21	60	33.0 > 3Grade: 2.0
ICI + SBRT	TIAN S [26]	2019	Clinical Trial	117	SBRT	33.9 > 3Grade: 10.7

*RP* Radiation pneumonitis, *SBRT* Stereotactic Body Radiotherapy, *RT* Radiation Therapy, *ICI* Immune checkpoint inhibitor

## Underlying medical conditions

In addition to the treatment factors related to RP summarized above, the patient's health and disease status is also closely associated with the occurrence of RP during treatment, such as age and sex, and other underlying diseases, such as interstitial lung disease [104], reduced lung function [105], diabetes and heart disease [106]. In addition, the occurrence of RP is also associated with nutritional status [107] and tumour volume [108, 109]. This section will describe the above risk factors.

### Age and sex

The relationship between age and sex and the occurrence of RP remains unclear. Most scholars have noted that old age is a high-risk factor for RP, which may be related to the fact that older patients often have additional underlying diseases and poor pulmonary function [33, 110]. Nevertheless, some scholars noted no significant correlation between age and concurrent chemoradiotherapy, but statistical bias caused by the small number of patients included in the group could not be excluded [111, 112]. Regarding sex, women are thought to be susceptible to RP, likely because their lung volume is smaller than that of men [113, 114].

### Interstitial lung disease (ILD)

Interstitial lung disease (ILD) has been reported as an independent risk factor for RP above grade 2 [105, 115]. In a retrospective analysis by Onishi et al., 72.2% of patients with fatal RP had higher than average KL-6 levels (elevated serum Krebs Von den Lungen-6 could indicate the presence of ILD), and more than 60% of patients with RP had CT findings of interstitial lung disease before treatment [112]. Coincidentally, a study by Sanuki et al. also showed that compared with patients without interstitial lung disease, the incidence of RP in patients with interstitial lung disease before treatment could be increased from 3 to 26% [33]. Therefore, patients with interstitial lung disease should be vigilant before receiving radiotherapy.

### Pulmonary function (PF)

Previous literature has confirmed that the incidence of RP is higher in patients with poor PF for the same dose of radiation, and PF may be an independent predictor of RP [116–118]. Wang et al. showed that the incidence of RP in patients with FVC less than 2.41 L was as high as 50% [119]. Therefore, PF is of great significance in the high-risk population of RP. We recommend routine PF examination before radiotherapy.

### Diabetes

Many studies have shown that diabetes is a critical risk factor for RP [120–123]. On the one hand, pathophysiological processes, such as inflammatory reactions, oxidative stress, and microvascular disease caused by a high blood glucose, are very similar to the pathophysiological changes of RP. On the other hand, vascular disease caused by high glucose also blocks the repair of tissue radiation damage.

### Heart disease

Few studies have directly confirmed the relationship between RP and cardiovascular diseases, such as hypertension and coronary arteriosclerotic heart disease. Van Luijk et al. noted that the increase in cardiac dose will significantly affect the incidence of lung-related adverse events, which may be due to the superposition of cardiopulmonary injuries [124]. In addition, an animal experiment also showed that co-irradiation of the heart and lung would cause the superposition of heart and lung injury, and the incidence of adverse events of both would increase [125].

### Nutritional status

RP can occur during radiotherapy until half a year after the end of radiotherapy, and this long period and the high nutritional cost of tumours easily cause malnutrition in late-stage patients. Immune deficiency caused by malnutrition may make patients more susceptible to infection. Therefore, we suggest that patients receiving radiotherapy should pay attention to their nutritional status, which may be a simple method to control RP. Ma et al. included 150 patients in a study on nutritional status and RP. The results showed that 40% of patients with malnutrition had RP, while patients with good nutritional status had no RP [107].

## Conclusion

In summary, the occurrence of RP is closely related to the physical parameters of radiotherapy and systemic therapy, including synchronous chemotherapy, immunotherapy, and targeted therapy. In addition, the nutritional status of patients and the associated basic diseases also impact the generation of RP. With the advent of the era of immunotherapy and drugs aimed at new targets, treatment plans for lung cancer have also become diversified and individualized. However, radiotherapy continues to play an important role in the treatment of lung cancer. As a frequent complication of radiotherapy that dramatically affects the prognosis of patients, RP has permanently restricted clinicians from making treatment decisions. At present, only symptomatic treatment is available for RP, and unified and effective intervention and prevention measures are lacking.

The purpose of this literature review is to provide a comprehensive review of risk factors that significantly increase the risk of RP. We hope to provide a reference for radiation therapists to prevent, avoid or reduce the occurrence of these events. Hopefully, these measures will improve the quality of life and prognosis of patients and more effectively improve the therapeutic effect of radiation therapy. Moreover, the description of the mechanism of RP also provides guidance to seek effective strategies to prevent RP in the future.

Notably, the production of oxidative free radicals and further oxidative damage can increase the infiltration of inflammatory cells. Berberine was previously reported as a protective agent against oxygen-free radicals to inhibit damage, and this result was only demonstrated in liver toxicity. We wanted to investigate whether a similar protective effect could be achieved in lung damage [126]. Finally, we are willing to continue exploring and forging ahead in this direction to find a definitive answer.

## Data Availability

The dataset supporting the conclusions of this article is included within the article.

## Abbreviations

RP: Radiation pneumonitis
DVH: Dose-volume histogram
MLD: Mean lung dose
NTCP: Normal tissue complication probability
EGFR-TKI: Epidermal Growth Factor Receptor-Tyrosine Kinase inhibitor
NSCLC: Non-Small Cell Lung Cancer
ALK: Anaplastic Lymphoma Kinase
3D-CRT: 3-Dimensional Conformal Radiation Therapy
SBRT: Stereotactic Body Radiotherapy
PTV: Planning Target Volume
RT: Radiation Therapy
PD-L1: Programmed Cell Death-Ligand 1 inhibitor
PD-1: Programmed Cell Death 1 inhibitor
PD-L2: Programmed Cell Death-Ligand 2 inhibitors
PFS: Progression-Free Survival
OS: Overall Survival
ORR: Objective Response Rate
VEGF: Vascular Endothelial Growth Factor
VEGFR: Vascular Endothelial Growth Factor Receptor
FGFR: Fibroblast Growth Factor Receptors
ILD: Interstitial lung disease
PF: Pulmonary function
